# Disabilities in children receiving care and support in Wales and risk factors associated with being placed in care: A population-based data linkage study

**DOI:** 10.23889/ijpds.v9i5.2605

**Published:** 2024-09-18

**Authors:** Jeanne Childs, Lucy Griffiths, Helen Hodges, Grace Bailey, Martin Elliott, Laura Cowley

**Affiliations:** 1 Swansea University; 2 Cardiff University

## Objectives

To determine the prevalence of functional impairments in children with disabilities receiving care and support in Wales between 2016 and 2022, and to investigate risk factors associated with being placed in care amongst these children.

## Method

This study used Children Receiving Care and Support (CRCS) Census records held in the Secure Anonymised Information Linkage Databank. We examined the proportion of children with specific impairments by their looked-after status, using bar charts and Upset plots. CRCS records were linked to demographic datasets to obtain information on area-level deprivation, and we conducted multivariable logistic regression analyses to model risk factors associated with being placed in care for children with disabilities.

## Results

Of 38,165 children receiving care and support, 10,075 (26%) had a disability, and 3,360 (33%) of these children were placed in care. Older children were less likely to be placed in care than infants (e.g. for age group 1‒4: OR 0.37 (95% CI 0.24‒0.56)). Children of mixed ethnicity were more likely to be placed in care compared to White children (2.17 (1.65‒2.85)). Child mental health problems were associated with being placed in care (1.82 (1.60‒2.07)) as were parental mental health problems (1.44 (1.29‒1.60)), parental substance/alcohol misuse (3.16 (2.81‒3.56)), parental learning disability (2.92 (2.51‒3.38)), and domestic abuse (1.48 (1.31‒1.66)).

## Conclusions

This new evidence may assist in the planning and provision of appropriate ongoing care and support for these children and their families to help prevent entry into the care system.

